# An essential role for the Ino80 chromatin remodeling complex in regulation of gene expression during cellular quiescence

**DOI:** 10.1007/s10577-023-09723-x

**Published:** 2023-04-12

**Authors:** Yasaman Zahedi, Shengyuan Zeng, Karl Ekwall

**Affiliations:** Department of Biosciences and Nutrition, Karolinska Institutet, Neo Building, 141 83 Huddinge, Sweden

**Keywords:** Cellular quiescence, fission yeast, chromatin remodelling, histone variant, Ino80, H2A.Z, eviction, telomere, boundary element

## Abstract

Cellular quiescence is an important physiological state both in unicellular and multicellular eukaryotes. Quiescent cells are halted for proliferation and stop the cell cycle at the G_0_ stage. Using fission yeast as a model organism, we have previously found that several subunits of a conserved chromatin remodeling complex, Ino80C (INOsitol requiring nucleosome remodeling factor), are required for survival in quiescence. Here, we demonstrate that Ino80C has a key function in the regulation of gene expression in G_0_ cells. We show that null mutants for two Ino80C subunits, Iec1 and Ies2, a putative subunit Arp42, a null mutant for the histone variant H2A.Z, and a null mutant for the Inositol kinase Asp1 have very similar phenotypes in quiescence. These mutants show reduced transcription genome-wide and specifically fail to activate 149 quiescence genes, of which many are localized to the subtelomeric regions. Using spike in normalized ChIP-seq experiments, we show that there is a global reduction of H2A.Z levels in quiescent wild-type cells but not in *iec1∆* cells and that a subtelomeric chromosome boundary element is strongly affected by Ino80C. Based on these observations, we propose a model in which Ino80C is evicting H2A.Z from chromatin in quiescent cells, thereby inactivating the subtelomeric boundary element, leading to a reorganization of the chromosome structure and activation of genes required to survive in quiescence.

**Supplementary information:**

The online version contains supplementary material available at 10.1007/s10577-023-09723-x.

## Introduction

Cellular quiescence is a reversible dormant state in which cells are changing their metabolism and cytology to adapt to an environment that does not permit proliferation. The ability to exit the cell cycle and enter quiescence is essential for tissue development and homeostasis in multicellular organisms. It is also an important survival strategy for unicellular organisms in harsh conditions. When fission yeast, *Schizosacharomyces pombe*, cells are starved for nitrogen in the absence of cells of the opposite mating type, they stop dividing and enter a quiescent state. In this state, the cell cycle is halted at the G_0_ stage before DNA replication. In G_0_, cells adapt the metabolism to survive until a nitrogen source becomes available so that the cells can re-enter the cell cycle and start proliferating. The survival in quiescence depends on a global change in gene expression (Marguerat et al. 2012). RNA transcription is generally reduced; however, some genes need to be activated to cope with the new physiological situation, for example, authophagy genes, proteasome-encoding genes, and genes for hexose and amino acid transporters (Takeda et al. 2010) (Oya et al. 2019). The nuclei of quiescent fission yeast cells are dramatically reorganized presumably to accommodate these profound changes in gene expression (Sajiki et al. 2009). To gain more insight into the role of chromatin structure changes in this process, we conducted a genetic screen in fission yeast for genes required to maintain viability during cellular quiescence (Zahedi et al. 2020). The screen identified the Ino80 complex (Ino80C). Ino80 is the catalytic subunit of this large chromatin remodeling complex consisting of approximately 10–14 protein subunits depending on the species. In fission yeast Ino80C consists of the following core subunits: Ino80, Arp5, Arp8, Rvb1, Rvb2, Alp5 (Arp4), Act1, Ies2, Ies4, Ies6, and Taf14 (Tfg3), and the accessory subunits: Iec1, Hap2, Iec3, Iec5, and Nht1 (Hogan et al. 2010) (Shevchenko et al. 2008) (Shan et al. 2020). The Ino80C subunit Iec1 (Ino Eighty Complex subunit 1) in fission yeast is similar to the Ying-Yang 1 (YY1) subunit of the human Ino80C and the function of Iec1 is to recruit Ino80C to target genes (Hogan et al. 2010). We have previously shown that mutations in nine tested subunits of Ino80C: Ies6, Nht1, Iec1, Iec3, Tfg3, Arp8, Ies2, Ies4, and the putative Ino80C subunit Arp42, lead to quiescence mortality phenotypes (Zahedi et al. 2020). Mutations in Hap2, Iec1, Arp8, Iec3, Nht1, Arp5, Ies4, and Ies2 were recently shown to be short-lived in stationary phase in fission yeast, implicating Ino80C in chronological ageing (Romila et al. 2021). Thus, at least six Ino80C subunits: Iec1, Arp8, Iec3, Nht1, Ies4, and Ies2 are implicated both in survival in quiescence and chronological ageing.

The molecular function of Ino80C is to remove the histone variant H2A.Z from nucleosomes by a histone exchange mechanism with H2.A in a process driven by ATP hydrolysis (Papamichos-Chronakis et al. 2011). This function is important for the repair of double-strand breaks, DNA replication, and the regulation of transcription (Poli et al. 2017). The H2A.Z exchange mechanism may also involve RNA polymerase II (Pol II) activity (Ranjan et al. 2020). Ino80 has recently been reported to be involved in gene regulation in several different species. Ino80 is required to activate the transcription of genes involved in thermomorphogenesis in plants (Xue et al. 2021). This mechanism of gene activation by Ino80 in plants involves H2A.Z eviction in response to elevated temperature. In *Candida albicans*, Ino80 is required for hyphal development by H2A.Z eviction at hyphal genes (Zhao et al. 2022). In mouse embryonic stem cells, Ino80 is required for regulation of cell cycle transitions by activating cell cycle genes (Yoo et al. 2022). Hence, it is likely that the requirement for Ino80 during quiescence involves some aspect of transcription regulation.

In fission yeast, Ino80C was shown to be important also for histone H3 exchange (Singh et al. 2020). We found that the genes for H2A.Z (*pht1*) and histone H3 (*hht2*) are both essential for surviving quiescence suggesting that H2A.Z deposition or removal and new histone H3 expression are required to maintain viability in G_0_ (Zahedi et al. 2020). H2A.Z is deposited by the Swr1C complex (Swi2/Snf2-related ATPase) in fission yeast (Buchanan et al. 2009). However, mutations affecting Swr1C do not affect survival in quiescence, indicating that H2A.Z deposition in G_0_ is less important than its removal by Ino80C. The activity of Ino80C in budding yeast, *Saccharomyces cerevisiae*, is modulated by Inositol polyphosphates (Shen et al. 2003). Curiously, the inositol kinase, Asp1, was recently reported to be important for the survival of quiescence in fission yeast (Sajiki et al. 2018). To advance the understanding of the role of Inositol polyphosphates and histone exchange by Ino80C in fission yeast quiescence, here, we investigate the functions of Asp1, Iec1, Arp42, Ies2, H2A.Z, and histone H3 in the regulation of gene expression in G_0_ cells.

## Results

### Time course analysis of viability and RNA expression in G_0_

**Fig. 1:**
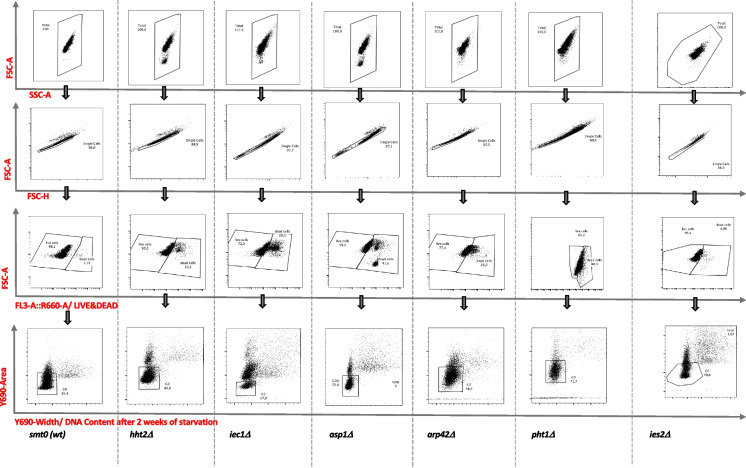
Measurements of viability and DNA content using flow cytometry. The gating strategy for measurement of viability and the proportion of G_0_ arrested cells during quiescence is illustrated with examples of FACS profiles for wild type (*smt0*) and the different mutant cells (as indicated)

We performed RNA sequencing (RNA-seq) analysis in wild-type cells and in three Ino80C-related mutants: *arp42∆*, *iec1∆*, and *ies2∆*, in *pht1∆* cells carrying a gene deletion for H2A.Z, in *asp1∆* cells carrying a gene deletion for an inositol kinase, and in *hht2∆* cells with a gene deletion for a histone H3 encoding gene. Cells were grown to the logarithmic phase in minimal medium and shifted to nitrogen-free minimal medium. Samples were taken for RNA extractions at time zero (T0, before shift) and after 24 h (T1D), 1 week (T1W), and/or 2 weeks (T2W) of nitrogen starvation. We measured the viability of the cultures at each time point using flow cytometry (Fig. 1, Table 1). All the cultures entered efficiently into G_0_, as judged from the percentage of cells 1C DNA content, within 24 h (T1D) from the shift to nitrogen-free medium (Table 2). Consistent with our previous observations the Ino80C mutants *iec1∆∆ arp42∆*, *ies2∆* and *pht1∆* showed a reduced viability after 1 week in G_0_ (Table 1). We also found that *asp1∆* cells lost their viability after 1 week in quiescence. This is also in agreement with a previous report (Sajiki et al. 2018). However, *hht2∆* cells displayed a milder mortality phenotype in quiescence, only showing reduced viability after 2 weeks.

**Table 1:** Viability measurements by FACS

Genotype	T0	T1D	T1w	T2w
*smt-0 (wt)*	99,9 $\pm$ 0,6	99,7 $\pm$ 0,2	98,8 $\pm$ 1,3	98,30 $\pm$ 0,3
*hht2∆*	98,0 $\pm$ 5,8	96,5 $\pm$ 2,5	95,1 $\pm$ 1,0	92,7 $\pm$ 2,8
*pht1∆*	96,0 $\pm$ 3,7	93,7 $\pm$ 0,3	85,0 $\pm$ 1,7	70,9 $\pm$ 11,5
*iec1∆*	96,8 $\pm$ 2,3	92,6 $\pm$ 1,4	85,1 $\pm$ 1,7	68,1 $\pm$ 0,8
*asp1∆*	99,5 $\pm$ 0,6	98,7 $\pm$ 0,1	95,1 $\pm$ 0,1	62,8 $\pm$ 3,7
*arp42∆*	99,4 $\pm$ 0,5	98,7 $\pm$ 0,1	87,2 $\pm$ 1,1	75,4 $\pm$ 2,7
*ies2∆*	*99,0; 99,6	*98,0; 98,2	*78,0; 82,5	*68,2; 65,1

The percentage of viable cells is indicated

Mean value $\pm$ standard deviation (*n* = 3)

^*^shows two measurements

**Table 2:** DNA content measurements by FACS

Genotype	T1D	T1w	T2w
*smt-0 (wt)*	81,9 $\pm$ 1,91	83,3 $\pm$ 3,23	85,0 $\pm$ 0,36
*hht2∆*	83,8 $\pm$ 1,07	86,4 $\pm$ 2,36	87,1 $\pm$ 2,51
*pht1∆*	61,4 $\pm$ 13,1	71,6 $\pm$ 13,4	74,5 $\pm$ 11,1
*iec1∆*	40,8 $\pm$ 11,5	40,8 $\pm$ 13,6	15,2 $\pm$ 0,42
*asp1∆*	67,3 $\pm$ 0,80	71,4 $\pm$ 0,57	74,9 $\pm$ 0,66
*arp42∆*	67,3 $\pm$ 0,85	71,9 $\pm$ 4,13	80,4 $\pm$ 1,51
*ies2∆*	*56,4; 56,9	*64,5; 64,8	*72,2; 70,6

The percentage of G_0_ cells is indicated (cells with a 1c DNA content)

Mean value $\pm$ standard deviation (*n* = 3)

^*^shows two measurements

### A global repression of the transcriptome in G_0_ and an induction of subtelomeric genes

**Fig. 2:**
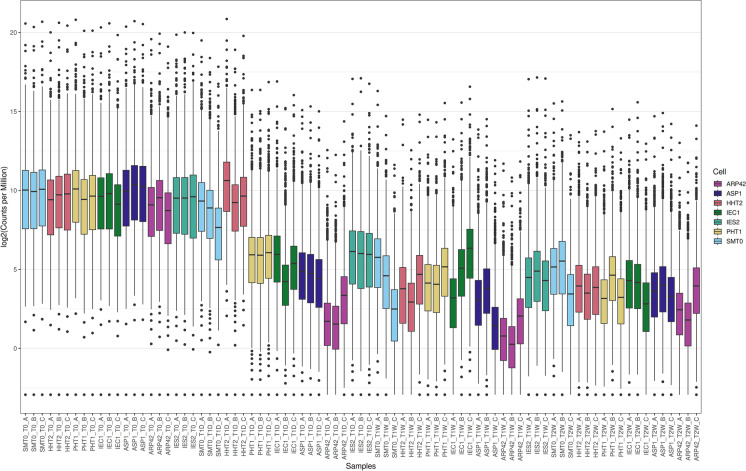
A representation of the ERCC spike in normalized RNA-seq data. The box plot shows triplicate RNA-seq samples as log2 values of numbers of normalized sequence reads (Y-axis) in wild-type cells (*smt0*) and the different mutants (as indicated in the X-axis)

For RNA extractions, we used biological triplicates for wild type and each mutant. Because we expected a global change of transcription in quiescence, we could not normalize the RNA-seq data to the total number of reads, since it would give false negative and false positive results. Instead, we normalized the data to external RNA control consortium (ERCC) spike in controls that were added in proportion to the number of cells in each sample (Risso et al. 2014). It was previously shown that the fission yeast transcriptome is strongly downregulated in quiescence (Marguerat et al. 2012). Consistent with the earlier study, the ERCC normalization clearly showed that gene expression was globally repressed in wild-type cells after 1 and 2 weeks in G_0_ (Fig. 2). The *hht2∆* cells showed a similar tendency as wild type. However, in *pht1∆*, *iec1∆∆*, *arp42∆*, *ies2∆*, and *asp1∆* cells, the global repression had occurred already after 24 h (Fig. 2).

**Fig. 3:**
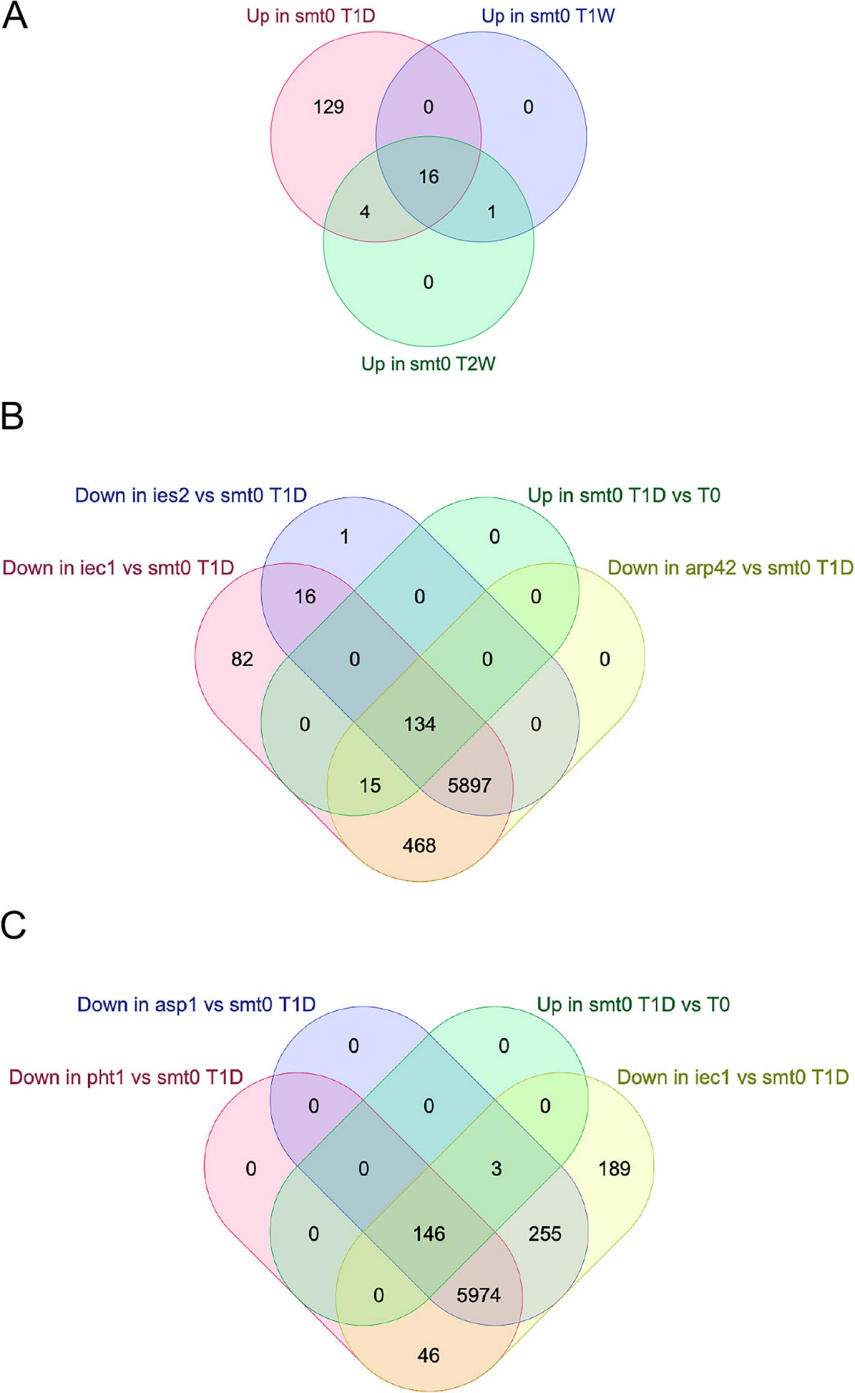
Analysis of gene expression changes during quiescence in wild type and Ino80C mutants. **A** Venn diagram comparing lists of genes upregulated in wild-type cells (*smt0*) at 24 h (T1D) 1 week (T1W) and 2 weeks (T2W) after removal of nitrogen. **B** Venn diagram comparing lists of genes downregulated in Ino80C mutants *iec1∆*, *ies2∆* and *arp42∆* with a list of genes upregulated in wild-type cells (*smt0*) at 24 h (T1D) after removal of nitrogen. **C** Venn diagram paring lists of genes downregulated in the Inositol kinase null mutant *asp1∆* with *iec1∆* and a null mutant for the histone variant H2A.Z (*pht1∆*) as well as the list of genes upregulated in wild-type cells (*smt0*) at 24 h (T1D) after removal of nitrogen

Next, a statistical threshold (FDR adjusted *P* < 0.05) was used to define up- and down-regulated genes. First, we defined genes affected by the shift to –nitrogen in wild type at T1D, T1W, and T2W compared to T0 (Table 3). These results are in nice agreement with earlier studies showing that most genes are downregulated and only a few genes are upregulated in G_0_. We compared the relatively few upregulated genes at the three time points in wild type (*smt0*) cells. There were 149 genes upregulated at T1D, 17 genes at T1W, and 21 genes at T2W. However, only 16 genes which were upregulated at all time points. Thus, we defined a set of 16 “core quiescence genes” that were found to be upregulated throughout the quiescence time course (Fig. 3A). Interestingly, 9 of these 16 genes (56.3%; CHI^2^ = 64; *P* < 0.001) reside in subtelomeric regions near *tel1R* and *tel2L* (Table 4). To validate the data, we compared with a previous quantitation of absolute numbers of mRNA molecules per cell (Marguerat et al. 2012). In all 16 cases, these measurements of mRNA molecules confirmed an induction of transcription in quiescent cells. Thus, our RNA-seq approach with ERCC normalization and a statistical cut off for affected genes was justified.

**Table 3:** Number of genes differentially expressed in quiescent wild-type cells compared to vegetative cells

Comparison	Up	Down	NS
T1D_T0_SMT0	149	1208	5255
T1W_T0_SMT0	17	6436	159
T2W_T0_SMT0	21	6360	231

The indicated gene lists were compared and the number of differentially expressed genes (up or down) are shown for each comparison (FDR adjusted *P* < 0.05)

RNA levels were normalized across all groups with ERCC spike-in controls

*NS*, not significant

**Table 4:** Features of 16 core quiescence genes

Geneid	Gene name	Quant. veg	Quant. G_0_	Gene localization
SPNCRNA.821		0.038	0.17	
SPAC22H10.13	*zym1*	2.7	17	
SPAC3G9.11c	*pdc201*	1.7	6.4	
SPAC1F7.06	*hsp3105*	0.023	1.2	
SPAC869.09		0.077	0.62	*subtel1R*
SPAC869.07c	*mel1*	0.069	0.66	*subtel1R*
SPAC869.06c	*hry1*	0	0.19	*subtel1R*
SPAC869.04		0.033	28	*subtel1R*
SPAC869.03c		0.025	10	*subtel1R*
SPBPB21E7.01c	*eno102*	0.11	0.4	*subtel2L*
SPBPB21E7.02c		0	0.12	*subtel2L*
SPBPB21E7.10		0	0.11	*subtel2L*
SPBPB21E7.11		0.13	0.49	*subtel2L*
SPNCRNA.1364		0.079	0.94	
SPNCRNA.1573		0.078	0.68	
SPBC2G2.17c		0.52	3.6	
SPNCRNA.577		0.016	4.6	

Quantitative data (number of molecules per cell) from Marguerat, S.; Schmidt, A.; Codlin, S.; Chen, W.; Aebersold, R.; Bähler, J., Quantitative analysis of fission yeast transcriptomes and proteomes in proliferating and quiescent cells. *Cell* 2012, *151*, 671–683

Next, we examined the 149 genes that were upregulated only at T1D, and a significant fraction of these genes are also localized close to telomeres. A total of 25 of the 149 genes (16.8%; CHI^2^ = 28,4; *P* < 0.001) are in 200 kb subtelomeric regions of chromosomes 1 and 2 (Table 5). Regarding the downregulated genes in G_0_, we found that as many as 1208 genes were significantly down after 24 h (T1D) and 6436 genes (including non-coding RNA genes) were down at T1W (Table 1). This represents 97.3% of the genome indicating that nearly the entire transcriptome is downregulated after 1 week in quiescence.

**Table 5:** Genomic features of 149 quiescence genes. Subtelomeric genes on chromosomes I and II (< 200 kb from ends) are in bold letters

Gene id	Chr	Start	End	Gene_name	T1_T0_SMT0_logFC	T1_T0_SMT0 *P*-value
**SPNCRNA.602**	**I**	**50851**	**51545**	**SPNCRNA.602**	**3,25973596**	**2,7274E-05**
**SPAC1F8.04c**	**I**	**92387**	**93931**	**SPAC1F8.04c**	**3,53511935**	**3,0867E-06**
**SPNCRNA.607**	**I**	**99046**	**101140**	**fta5-antisense-1**	**2,7031025**	**0,00024108**
**SPAC11D3.19**	**I**	**106893**	**108361**	**SPAC11D3.19**	**3,01020676**	**4,5012E-05**
**SPAC11D3.03c**	**I;I;I**	**110904;112098;112378**	**112046;112333;112499**	**SPAC11D3.03c**	**4,44873642**	**8,8882E-09**
**SPAC11D3.16c**	**I**	**140381**	**141653**	**SPAC11D3.16c**	**2,48746727**	**0,000418**
**SPAC11D3.17**	**I;I**	**141199;141559**	**141510;144768**	**SPAC11D3.17**	**2,1184002**	**0,00225435**
**SPAC13G6.08**	**I**	**187701**	**190031**	**SPAC13G6.08**	**2,11089891**	**0,00512897**
SPNCRNA.643	I	388352	389177	trm112-antisense-1	2,47596303	0,00370115
SPNCRNA.649	I	446175	446702	lsd2-antisense-1	2,79371868	0,00369267
SPAC23E2.03c	I	450860	453603	ste7	3,25111192	7,4801E-06
SPNCRNA.673	I	664677	665246	rad15-antisense-1	2,42711956	0,00934543
SPNCRNA.690	I	829234	831663	prh1-antisense-1	3,67766921	0,00038004
SPNCRNA.220	I	1015475	1015830	SPNCRNA.220	3,3952792	0,0028625
SPAC56F8.15	I	1151431	1153638	SPAC56F8.15	1,96273702	0,00753558
SPNCRNA.737	I	1357621	1359568	SPNCRNA.737	1,93983373	0,00875406
SPAC1002.19	I	1835270	1837060	urg1	3,37759044	3,9743E-06
SPAC1399.03	I	1841908	1843995	fur4	1,81787099	0,00812566
SPNCRNA.178	I	1844130	1845339	SPNCRNA.178	2,90780759	0,00018621
SPAP11E10.02c	I	1856833	1860727	mam3	2,58249425	0,00026631
SPAPB1A10.14	I	1890529	1892090	pof15	2,58471259	0,00031153
SPAC3C7.02c	I	2064853	2066004	pil2	2,01101362	0,00384745
SPNCRNA.791	I	2066217	2067343	rad55-antisense-1	3,26641538	7,9496E-05
SPAC20H4.11c	I;I	2131685;2132545	2132426;2133066	rho5	2,23485497	0,00175906
SPAC13F5.07c	I;I	2184654;2185472	2185282;2185990	hpz2	2,95890939	6,5014E-05
SPAC13G7.13c	I	2318492	2322664	msa1	2,01610833	0,00357637
SPNCRNA.821	I	2380416	2381234	SPNCRNA.821	5,22655651	2,8497E-07
SPAC22H10.13	I	2381690	2382200	zym1	5,19708373	9,4432E-11
SPNCRNA.194	I	2424735	2425515	SPNCRNA.194	2,33292483	0,00869259
SPNCRNA.43	I	2432340	2432921	prl43	3,36282112	0,00071322
SPAC4A8.04	I	2544815	2546981	isp6	2,06781376	0,00291872
SPNCRNA.835	I	2547093	2547449	SPNCRNA.835	2,20795116	0,0071483
SPSNRNA.06	I;I	2562276;2562374	2562323;2562427	snu6	1,87091948	0,00697876
SPNCRNA.198	I	2703965	2704030	SPNCRNA.198	2,78396522	0,00553514
SPNCRNA.853	I	2784605	2785492	SPNCRNA.853	4,11372383	3,3265E-07
SPNCRNA.860	I	2931622	2932800	SPNCRNA.860	5,01730583	3,0063E-09
SPAC2E1P3.04	I	2931763	2935115	cao1	2,22542246	0,00144898
SPAC31G5.09c	I	2999546	3001687	spk1	1,84222622	0,00732841
SPNCRNA.71	I	3001761	3001930	SPNCRNA.71	2,61362541	0,00116616
SPNCRNA.877	I	3032579	3033397	SPNCRNA.877	2,8566656	0,00067403
SPAC24C9.16c	I;I;I;I	3042749;3042872;3043096;3043225	3042817;3042909;3043157;3043380	cox8	1,88137663	0,00786675
SPAC3G9.11c	I	3159753	3162245	pdc201	4,74996563	1,3887E-09
SPAC6G10.06	I;I;I	3226929;3227042;3227456	3226980;3227383;3228192	SPAC6G10.06	2,51110956	0,0013486
SPNCRNA.931	I	3731313	3732279	grx2-antisense-1	2,91994619	0,0014984
SPNCRNA.935	I	3745778	3748833	SPNCRNA.935	3,9191636	1,7498E-07
SPATRNAGLU.04	I	3776768	3776839	SPATRNAGLU.04	1,95115116	0,01020029
SPNCRNA.955	I	4004553	4005632	SPAC27E2.02-antisense-1	2,33505779	0,00572603
SPSNORNA.13	I	4154912	4155002	snoR69b	2,03181845	0,00326288
SPAC25B8.13c	I	4179961	4183074	isp7	2,4050579	0,00062889
SPAC1F7.06	I	4230615	4232557	hsp3105	5,80756074	2,7966E-11
SPNCRNA.243	I	4231812	4232976	SPNCRNA.243	2,57917641	0,00141246
SPAC9E9.17c	I;I;I;I	4437311;4437606;4437732;4437860	4437401;4437648;4437813;4437862	SPAC9E9.17c	5,21541272	8,4446E-09
SPNCRNA.987	I	4437466	4438921	SPAC9E9.17c-antisense-1	2,17485116	0,00635584
SPNCRNA.12	I	4439118	4439451	prl12	3,78490716	0,00470787
SPAC17C9.16c	I;I	4472472;4473325	4473271;4474744	mfs1	1,83115795	0,00796667
SPAC27D7.03c	I	4510983	4515015	mei2	2,22978588	0,00141295
SPNCRNA.993	I	4515182	4515993	SPNCRNA.993	2,45668478	0,00117438
SPAC11H11.04	I	4780224	4781922	mam2	4,13282167	4,892E-08
SPAC4F10.17	I	4868326	4869283	SPAC4F10.17	3,91344782	1,839E-06
SPAC4F10.22	I	4878241	4878453	cmc4	2,0237062	0,00648228
SPAPB8E5.04c	I	4913971	4915513	npc2	1,84500451	0,00745054
SPAPB8E5.05	I;I;I	4916603;4916921;4917286	4916841;4917214;4917521	mfm1	7,30766685	1,2375E-16
SPAPJ691.02	I	5187647	5189187	SPAPJ691.02	4,53526941	4,6374E-09
SPAC19D5.07	I	5222447	5224795	uga1	1,88556666	0,00622319
SPAC2H10.01	I	5273012	5275983	SPAC2H10.01	2,6493213	0,00027561
SPNCRNA.1068	I	5309027	5311689	SPNCRNA.1068	2,30679984	0,00316561
SPNCRNA.1075	I	5353770	5354310	new12-antisense-1	3,31258143	9,0267E-05
**SPAC1039.03**	**I**	**5452105**	**5453867**	**SPAC1039.03**	**2,12458926**	**0,00219715**
**SPAC1039.09**	**I**	**5465377**	**5468649**	**isp5**	**2,15393594**	**0,00202143**
**SPAC1039.10**	**I;I;I**	**5470432;5470574;5471284**	**5470525;5471227;5471520**	**mmf2**	**1,86772985**	**0,00694076**
**SPAC922.09**	**I**	**5482662**	**5482877**	**SPAC922.09**	**5,12304127**	**4,3744E-05**
**SPAC922.06**	**I**	**5485096**	**5486287**	**SPAC922.06**	**2,20990467**	**0,00153079**
**SPNCRNA.1092**	**I**	**5495270**	**5496597**	**SPNCRNA.1092**	**2,16976747**	**0,00364619**
**SPAC869.09**	**I**	**5496844**	**5497448**	**SPAC869.09**	**4,67744479**	**8,7417E-06**
**SPAC869.08**	**I**	**5497918**	**5499280**	**pcm2**	**4,23373637**	**4,4033E-07**
**SPAC869.07c**	**I**	**5499768**	**5501236**	**mel1**	**4,70824039**	**6,0466E-09**
**SPAC869.06c**	**I**	**5502560**	**5503291**	**SPAC869.06c**	**3,4837771**	**0,00042619**
**SPAC869.04**	**I**	**5511137**	**5512369**	**SPAC869.04**	**9,23276947**	**6,5934E-22**
**SPAC869.03c**	**I**	**5512757**	**5514865**	**SPAC869.03c**	**9,16835946**	**9,8841E-22**
**SPAC869.01**	**I**	**5521275**	**5523181**	**SPAC869.01**	**3,00278941**	**0,00011382**
**SPBPB21E7.02c**	**II;II**	**60553;61119**	**61107;61205**	**SPBPB21E7.02c**	**10,5415628**	**0,00031848**
**SPBPB21E7.10**	**II;II**	**61362;61632**	**61526;62126**	**SPBPB21E7.10**	**10,3509696**	**0,00048232**
**SPBPB21E7.11**	**II;II;II**	**61526;62598;62944**	**62449;62885;63086**	**SPBPB21E7.11**	**4,55305877**	**2,1632E-06**
**SPBC1683.05**	**II**	**147915**	**150574**	**SPBC1683.05**	**1,81584673**	**0,00821874**
SPBC1271.09	II;II	350692;350787	350740;352369	tgp1	2,42462905	0,00053769
SPNCRNA.1346	II	359391	359866	SPNCRNA.1346	3,2706138	0,00044942
SPSNRNA.04	II	467233	467361	snu4	3,23066699	0,00022388
SPNCRNA.1364	II	503961	505097	SPNCRNA.1364	4,8789878	5,6148E-10
SPBC1685.05	II	504397	509056	SPBC1685.05	2,22522485	0,0014316
SPNCRNA.66	II	547325	547761	prl66	2,67117747	0,0054103
SPBC354.12	II;II	578063;578725	578181;580056	gpd3	2,77080717	9,979E-05
SPBPJ4664.03	II;II	700291;700544	700476;700700	mfm3	7,90991958	3,2907E-18
SPNCRNA.1405	II	1013315	1014054	prp2-antisense-1	3,05365283	0,00040845
SPNCRNA.1415	II	1150424	1150863	SPNCRNA.1415	6,49255957	7,8126E-08
SPSNORNA.21	II	1308634	1308745	snoU14	3,06732048	1,982E-05
SPNCRNA.15	II	1329046	1329760	prl15	7,65836499	9,1712E-11
SPBC83.19c	II	1541452	1541903	SPBC83.19c	11,1826139	4,0742E-05
SPNCRNA.352	II	1542196	1542364	SPNCRNA.352	11,0312641	6,4796E-05
SPBC29B5.02c	II	1550293	1553467	isp4	2,76293742	0,00010681
SPNCRNA.1443	II	1554843	1557638	SPNCRNA.1443	2,04831502	0,00323332
SPBC1D7.02c	II	1752100	1755619	scr1	2,04685207	0,0032455
SPBC9B6.03	II	1817454	1819848	SPBC9B6.03	2,88027667	5,562E-05
SPBC23G7.11	II	2120231	2121050	mag2	2,67196421	0,00085483
SPNCRNA.1512	II	2417501	2418682	pvg3-antisense-1	3,40867882	0,00292084
SPNCRNA.1525	II	2543249	2544500	psm1-antisense-1	3,50755144	1,6382E-05
SPNCRNA.1530	II	2580400	2581182	SPNCRNA.1530	9,90488058	0,0021415
SPBC25B2.08	II	2611366	2612917	SPBC25B2.08	2,18518869	0,00197078
SPBC19C7.04c	II	2825251	2826700	SPBC19C7.04c	2,71034397	0,00015799
SPNCRNA.1564	II	2934727	2935979	SPBC1703.11-antisense-1	2,52684517	0,00180399
SPNCRNA.1573	II	3020526	3022271	SPBC15D4.05-antisense-1	6,22415483	4,9082E-12
SPNCRNA.413	II	3150075	3150475	SPNCRNA.413	2,82207927	0,00847413
SPBC13A2.04c	II	3405575	3408492	ptr2	2,25820836	0,00121891
SPBC2G2.17c	II	3466270	3467797	SPBC2G2.17c	5,69625442	5,1176E-12
SPBC887.16	II	3574869	3575250	SPBC887.16	2,87571225	0,00131414
SPSNORNA.27	II	3654333	3654416	snoR47	2,08042095	0,0026702
SPNCRNA.1660	II	4049029	4049473	SPBC215.10-antisense-1	2,32255749	0,00367613
SPBC1347.11	II	4081869	4083198	sro1	2,14480969	0,00202154
SPNCRNA.1670	II	4109027	4109600	SPNCRNA.1670	5,26102836	1,1998E-06
SPNCRNA.577	II	4255755	4259164	SPBC1652.02-antisense-1	5,33538848	2,9985E-11
SPNCRNA.451	III	49802	50142	SPNCRNA.451	4,52899464	1,8957E-08
SPCC757.13	III	77080	79816	SPCC757.13	2,56643263	0,00028782
SPNCRNA.1126	III	289992	293300	SPCC553.08c-antisense-1	2,13247264	0,00505195
SPNCRNA.1137	III	394357	396657	ubp16-antisense-1	3,60875293	7,5933E-06
SPCC1183.12	III;III	604366;604857	604805;604961	spo13	2,01450177	0,00823742
SPNCRNA.1160	III	815167	816476	SPCC1393.09c-antisense-1	3,32393748	0,00134978
SPCPB16A4.06c	III;III	956012;957195	957014;957627	SPCPB16A4.06c	1,91007479	0,00587734
SPCC550.07	III	1196729	1199999	SPCC550.07	3,43129476	2,8671E-06
SPCC550.10	III	1204315	1206695	atd3	2,60470778	0,00022774
SPCC338.18	III	1340628	1341847	SPCC338.18	2,64277685	0,00033584
SPCC1281.04	III	1386524	1387961	SPCC1281.04	2,38484333	0,00223696
SPCC188.12	III;III	1504862;1505062	1504999;1506851	spn6	4,13670216	1,5614E-07
SPNCRNA.51	III	1514062	1514343	prl51	3,34790704	0,00037929
SPCC584.13	III	1516197	1518230	SPCC584.13	2,22673511	0,00137358
SPNCRNA.1205	III	1559743	1561240	SPNCRNA.1205	5,35400218	1,6137E-05
SPCC417.02	III	1669944	1671551	dad5	4,36062922	1,3103E-08
SPNCRNA.1215	III	1671883	1674238	SPCC417.03-antisense-1	2,29927601	0,00099408
SPCC417.06c	III;III;III	1677093;1678329;1678828	1678286;1678778;1679436	mug27	3,07341949	0,00197289
SPCC417.10	III	1694169	1697592	dal51	1,98405831	0,00416259
SPCC1450.07c	III;III	1736500;1738213	1737778;1738318	dao1	2,1507211	0,00212075
SPCC1442.01	III	1765913	1768971	ste6	2,32561278	0,00091354
SPCC1223.09	III	1857150	1858621	SPCC1223.09	2,18680094	0,00172594
SPNCRNA.1236	III	1859963	1860577	SPNCRNA.1236	1,78076961	0,00932741
SPCC74.04	III	1935474	1938754	SPCC74.04	2,86776786	6,048E-05
SPCC576.01c	III	2079421	2080662	xan1	4,08792138	6,3569E-08
SPCC830.04c	III	2186723	2187154	mug128	2,84134074	0,00746144
SPCC965.13	III	2310096	2314400	SPCC965.13	2,22721546	0,00139801
SPCC70.04c	III;III;III	2352568;2353746;2353974	2353701;2353913;2354572	SPCC70.04c	2,53908379	0,00031576
SPNCRNA.519	III	2362179	2362725	SPNCRNA.519	2,33386995	0,0013251
SPCC569.09	III;III	2413,905;2414158	2414089;2414790	SPCC569.09	274,408957	0,00012036

### Transcription changes in G_0_ and reduced viability of Asp1, Ino80C, and H2A.Z mutants

Next, we analyzed the changes of the G_0_ transcriptome in the mutants. It was clear already by looking at the total number of reads after ERCC normalization that all mutants showed overall changes in the G_0_ transcriptome as compared to wild type (Fig. 2). The overall tendency was that the mutants showed a further reduction of global transcription as compared to the wild type. The genes affected in the different mutants at each time point as compared to the wild type were defined (Table 6). This analysis revealed substantial changes in gene expression in all the tested mutants. Again, *hht2∆* cells showed a weaker phenotype at T1D and T1W compared to the other mutants, but 1286 genes were downregulated in *hht2∆* cells at T2W. The *hht2*^+^ gene is constitutively expressed in contrast to the other two histone H3-encoding genes (*hht1*^+^ and *hht3*^+^) in fission yeast which are strictly expressed during the S phase (Takayama and Takahashi 2007). Thus, *hht2*^+^ is the sole histone H3 gene expressed in G_0_ cells. The viability of *hht2∆* cells drops significantly compared to wild type after 2 weeks in quiescence (TTEST; *P* = 0.013) and is correlated with reduced transcription (Table 1, Fig. 2, Table 6). It is possible that this reduction of transcription, caused by reduced histone H3 levels, is contributing to the mortality of *hht2∆* cells in G_0_.

**Table 6:** Number of genes differentially expressed in mutant cells compared to wild-type cells

Comparison	Up	Down	NS
HHT2_SMT0_T0	2	6	6605
PHT1_SMT0_T0	5	9	6599
IEC1_SMT0_T0	20	11	6582
ASP1_SMT0_T0	26	19	6569
ARP42_SMT0_T0	9	114	6490
IES2_SMT0_T0	9	29	6575
HHT2_SMT0_T1D	91	1 (hht2)	6521
PHT1_SMT0_T1D	6	6143	464
IEC1_SMT0_T1D	8	6194	410
ASP1_SMT0_T1D	2	6368	243
ARP42_SMT0_T1D	0	6519	94
IES2_SMT0_T1D	7	6048	558
HHT2_SMT0_T1W	1	1	6611
PHT1_SMT0_T1W	29	1	6583
IEC1_SMT0_T1W	88	0	6525
ASP1_SMT0_T1W	230	3504	2879
ARP42_SMT0_T1W	5	6145	463
IES2_SMT0_T1W	24	6	6583
HHT2_SMT0_T2W	190	1129	5294
PHT1_SMT0_T2W	16	33	6564
IEC1_SMT0_T2W	43	78	6492
ASP1_SMT0_T2W	144	1898	4571
ARP42_SMT0_T2W	5	5238	1370

The indicated gene lists were compared and the number of differentially expressed genes (up or down) are shown for each comparison (FDR adjusted *P* < 0.05)

RNA levels were normalized across all groups with ERCC spike-in controls

*NS*, not significant

In contrast to *hht2∆*, *pht1∆* cells show strong changes of the transcriptome already at 24 h, i.e., prior to the reduction in viability that occurs after 1 week (Fig. 1, Fig. 2, Table 6). This observation is consistent with a key role for the histone variant H2A.Z in gene regulation, being essential for survival in quiescence. Ino80C is required for the removal of H2A.Z by its chromatin remodeling activity driven by ATP hydrolysis (Papamichos-Chronakis et al. 2011). Our results show that null mutations in three Ino80C-related genes, *arp42∆*, *iec1∆*, and *ies2∆* cause a massive reduction of the G_0_ transcriptome after 24 h as compared to the wild type, and *arp42∆* shows a further reductions of gene expression after one and 2 weeks. Thus, a vast majority of genes are prematurely downregulated in Ino80C mutants compared to wild type (Fig. 2, Table 6). Although the observed transcription patterns are not strictly correlated to mortality, the generally reduced transcription is likely explaining the essential role of Ino80C in cellular quiescence that we previously observed (Zahedi et al. 2020).

### Failure to induce quiescence genes in Ino80C mutants

Next, we studied the behavior of the larger set of 149 genes that are upregulated in the wild type after 24 h in nitrogen starvation (T1D). We found that none of these genes were upregulated in *arp42∆*, *iec1∆*, and *ies2∆* cells (Fig. 3B). In fact, all the 149 genes were downregulated in these mutants compared to wild type (*smt0*) after 24 h in G_0_. This shows that Ino80C is essential for the activation of these genes in response to nitrogen starvation. Gene ontology analysis revealed that the list of 149 upregulated genes is significantly enriched for several GO terms including the fungal vacuole, amino acid, dipeptide, and nucleobase transmembrane transport (Table 7). The vacuole is required in quiescence for the autophagy process to recycle amino acids, and these transmembrane transport processes are crucial for survival during cellular quiescence. Thus, the upregulated expression of these genes is required for survival in G_0_ by adapting the cellular metabolism. For example, the *SPAC11D3.16c* gene located in near *tel1L* is annotated as being essential for viability in G_0_ (Harris et al. 2021). Two genes near *tel1R* encoding membrane transporters, *isp5*^+^ and *SPAC869.03c* fail to be activated in Ino80C mutants. Therefore, it is conceivable that the reason Ino80C mutants are dying in G_0_ is due to the inability to activate genes needed for the metabolic change and cellular uptake that normally occur during quiescence.

**Table 7:** Gene ontology analysis of 149 genes induced in quiescent wild-type cells after 24 h

GO	Term	N	Gene list T1D_T0_SMT0_up	*P*-value
GO:0,000,750	pheromone-dependent signal transduction involved in conjugation with cellular fusion	14	5	9,23456E-06
GO:0,035,442	dipeptide transmembrane transport	3	3	1,12193E-05
GO:0,071,916	dipeptide transmembrane transporter activity	3	3	1,12193E-05
GO:0,043,864	indoleacetamide hydrolase activity	3	2	0,001,491,016
GO:0,015,205	nucleobase transmembrane transporter activity	3	2	0,001,491,016
GO:0,007,267	cell–cell signaling	4	2	0,002,937,898
GO:0,000,772	mating pheromone activity	4	2	0,002,937,898
GO:0,000,324	fungal-type vacuole	68	6	0,004,105,498
GO:0,035,673	oligopeptide transmembrane transporter activity	5	2	0,004,824,158
GO:0,003,333	amino acid transmembrane transport	17	3	0,006,047,446
GO:0,031,520	plasma membrane of cell tip	20	3	0,009,650,323
GO:0,006,878	cellular copper ion homeostasis	9	2	0,016,367,608

Gene Ontology (GO) analysis of GO terms obtained from PomBase

Analysis done on the upregulated genes of the wild type (SMT0) cells between T0 and T1D

N column, the number of genes in the GO

### A role of Ino80 and H2A.Z in activation of quiescence genes

To get some insights into the role of H2A.Z in quiescence, we compared the list of downregulated genes in the *pht1∆* mutant at T1D with the gene lists of Ino80C mutants, *arp42∆* and *iec1∆*, and a list of genes induced in the wild type at T1D. This comparison revealed that all of the 149 genes that are upregulated in wild type (*smt0*) at T1D fail to be induced in *pht1∆* cells lacking H2A.Z (Fig. 3C). Also, there is a very strong overlap genome-wide between genes downregulated in *pht1∆* cells and those downregulated in *arp42∆* and *iec1∆* mutants. A total of 5951 genes were downregulated in all three mutants. Based on this and our observations, we concluded that H2A.Z and Ino80C are both somehow required for the activation of genes induced in G_0_, in particularly in subtelomeric regions.

### Changes of H2A.Z localization in quiescent cells at subtelomeric LTR boundary elements

**Fig. 4:**
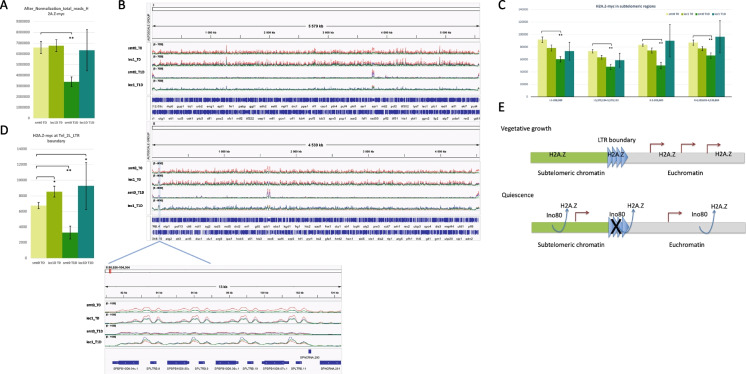
ChIP-seq analysis of H2A.Z localisation at a subtelomeric boundary element in vegetative (T0) and quiescent cells (T1D). **A** Quantitation of total number oif ChIP-seq reads for H2A.Z-myc. The bar diagram shows the total number of reads (after spike-in normalization) in wild-type cells (*smt0*) and the *iec1∆* mutant at the vegetative stage (T0) and 24 h after removal of nitrogen (T1D). The error bars represent Standard deviation (SD) values from triplicate samples. Unpaired t-test was used to determine data significance. *P < 0.05, **P < 0.01*.* **B** Browser images of chromosomes I, II and the LTR boundary element at *tel2L.* The IGV genome browser tracks present chromosome 1, chromosome 2 and LTR boundary at chromosome II subtelomeric region (chrII: 93,039–102,657). **C** Quantitation of ChIP-seq reads for H2A.Z-myc in subtelomeric regions. The bar diagram shows the reads in 4 subtelomeric region, chrI:1–200,000, chrI:5,379,134–5,579,133, chrII:1–200,000 and chrII:4,339,805–4,539,804. The error bars represent SD values from triplicate samples. Unpaired t-test was used to determine data significance. **P < 0.01*.* **D** Quantitation of ChIP-seq reads for H2A.Z-myc the LTR boundary element at *tel2L*. The bar diagram shown the reads in LTR boundary element at chromosome II subtelomeric region (chrII: 93,039–102,657). The error bars represent SD values from triplicate samples. Unpaired t-test was used to determine data significance. *P < 0.05, **P < 0.01*.* **E** Model for regulation of the activity of the *tel2L* boundary element. For details see Discussion

The molecular function of Ino80C is to remove H2A.Z in a nucleosome disassembly mechanism in which H2A.Z is exchanged with H2A (Papamichos-Chronakis et al. 2011). Furthermore, Ino80 has been shown to evict H2A.Z in diverse organisms leading to changes in gene expression. To test if the observed changes in gene expression in quiescent cells depend on H2A.Z, we performed ChIP-seq of epitope tagged H2A.Z (*pht1-myc*) in wild type and *iec1∆* cells. We used the *Drosophila* spike in chromatin methodology to allow measurement of global changes of H2A.Z occupancy by ChIP-seq (see the “Materials and methods” section). The total number of matched reads after the spike in normalization revealed a strong and significant reduction of H2A.Z-myc occupancy in quiescent wild type (*smt0*) cells at T1D compared to vegetative wild-type cells (T0), but not redusction was observed in quiescent *iec1∆* cells (Fig. 4A). The chromosomal browser view confirmed this observation showing very low signals of H2A.Z-myc along all three chromosomes in wild-type cells at T1D, whereas signals remained high in *iec1∆* cells (Fig. 4B). Thus, we conclude that there is an eviction process of H2A.Z from chromsosomes in quiescent cells, which is dependent on Ino80C.

Next, we examined the subtelomeric regions for changes of H2A.Z localization in quiescent cells (Fig. 4C). As illustrated by the bar diagrams subtelomeric regions (0–200 kb) of chromosomes I and II had a significant reduction (TTEST; *P* < 0.01) of H2A.Z in wild-type cells at T1D. Again this reduction was not observed in *iec1∆* cells at T1D. Furthermore, it was clear that H2A.Z is localized in four peaks to the long terminal repaet (LTR) containing subtelomeric boundary element near *tel2L* (Fig. 4B; bottom). The activity of this boundary element has previously been shown to be maintained via Fft3 (Fun thirty homolog 3) in vegetative cells (Strålfors et al. 2011) (Steglich et al. 2015). In vegetative cells (T0), we observed a small but significant increase of H2A.Z at the *tel2L* boundary in *iec1∆* cells compared to wild type (Fig. 4D). However, in quiescent wild-type cells, the H2A.Z peaks were strongly reduced (TTEST; *P* < 0.01) whereas the peaks could still be detected in quiescent *iec1∆* cells (Fig. 4D). It was not possible to investigate if Ino80C also plays a role at the he other subtelomeric boundary elements since they are not cleary defined (Steglich et al. 2015). Taken together, this suggests that Ino80 is involved in the eviction or relocalization of H2A.Z genomewide, in subtelomeric regions of chromosomes I and II, and at a subtelomeric LTR boundary element (*tel2L*) especially during quiescence.

## Discussion

### A role for Ino80 in quiescence and ageing?

We have shown that Ino80C is required for the expression of genes in quiescence, including the *hsp3105*^+^ gene, encoding a ThiJ domain protein implicated in autophagy and oxidative stress resistance (Table 4). This gene was shown to be required for survival in the stationary phase (Su et al. 2015). Assuming that Ino80C drives expression of *hsp3105*^+^ in the stationary phase, then it could explain the short chronological lifespan phenotype that was reported for Ino80C mutants (Romila et al. 2021). It would also suggest that there is some commonality between chronological ageing in stationary phase and quiescence.

### A possible role for Inositol polyphosphates restricting Ino80C activity in fission yeast?

Asp1 encodes an inositol kinase that may affect the activity of Ino80C. Inositol polyphosphates are synthesized by a series of enzymes including Asp1. The Asp1 kinase generates one specific inositol polyphosphate, IP_8_, and in fission yeast *asp1∆* mutants, IP_8_ levels are strongly reduced whereas IP_6_ and IP_7_ levels are increased (Pascual-Ortiz et al. 2018). In budding yeast IP_6_ directly inhibits Ino80C (Shen et al. 2003). Thus, our results showing a similar effect on the G_0_ transcriptome and G_0_ mortality phenotypes between *asp1∆* and Ino80C mutants and the strong overlap of downregulated genes (Fig. 3B) suggest that inositol polyphosphates IP_6_ and IP_7_, which accumulate in the *asp1∆* mutant, may inhibit Ino80C activity also in fission yeast.

### A change in nuclear organization in quiescence mediated by Ino80C?

In fission yeast, all four telomeres of chromosomes I and II, i.e., *tel1L*, *tel1R*, *tel2L*, and *tel2R*, form a peripheral cluster near the nucleolus in quiescent cells (Maestroni et al. 2020). It was previously shown that genes induced during meiosis and sporulation are enriched in subtelomeric regions (Mata et al. 2002). It is conceivable that Ino80C is involved in the formation of a transcriptionally active nuclear compartment comprising subtelomeres in response to nitrogen starvation or cellular quiescence when a mating partner is absent. Expression of this active compartment may support for mating and sporulation if cells of the opposite mating type are present. In agreement with this notion, the *iec1∆* mutant was reported to show a decreased mating efficiency (Hogan et al. 2010). In budding yeast, Ino80C and its ATPase activity are required for chromosomal movements within the nucleus (Neumann et al. 2012). Hence, it is possible that the formation of this actively transcribed subtelomeric nuclear compartment, in response to nitrogen starvation, involves chromatin movements facilitated by Ino80C within the nucleus.

Based on our new results, we propose a model in which Ino80C activity, possibly modulated by inositol kinase Asp1, is required to remove H2A.Z from chromatin by a nucleosome disassembly mechanism in quiescent cells (Fig. 4E). This includes H2A.Z eviction at a subtelomeric boundary element leading to inactivation of the boundary and gene expression of subtelomeric genes, including transmembrane transporter genes, required to survive in quiescence. We hypothesize that this process may involve a drastic reorganization of chromosome structures in quiescent cells and clustering of telomeres to maintain an active nuclear compartment. Interestingly, it is known that *fft3∆* cells have a reduced efficiency of to enter and exit quiescence (Sajiki et al. 2018; Zahedi et al. 2020). This is probably due to a failure in maintaining and restoring the subtelomeric boundary elements during these cellular transitions. To speculate further, the activation of quiescence genes by Ino80C is likely linked to the observed reduction of subtelomeric heterochromatin regions that occurs in quiescent cells (Oya et al. 2019). Consistent with this notion, it is known from studies in budding yeast that H2A.Z incorporation into acetylated chromatin by the SWR1-C complex is maintains heterochromatin boundary activity at silenced *HMR* loci and near telomeres (Zhou et al. 2010). Hence, in two distinct yeast species, H2A.Z is involved in maintaining heterochromatin boundaries.

In *Drosophila*, insulator boundary elements bound by the CTCF protein play important roles during development by partitioning the genome into distinct topologically associating domains (TADs), for example, in the *Antennopedia* gene complex where they prevent inappropriate enhancer promoter interactions between TADs (reviewed by (Batut et al. 2022)). The fission yeast genome is also organized into cohesion dependent TAD-like structures in vegetative cells (Mizuguchi et al. 2014). It is therefore tempting to speculate that the subtelomeric TAD structures are drastically reorganized in quiescent fission yeast cells is response to H2A.Z removal by Ino80. Finally, H2A.Z was recently implicated in regulating CTCF binding to chromatin by modulating the unwrapping of nucleosomes in mouse ES cells (Wen et al. 2020). Yeast cells do not have a CTCF protein; however, it is plausible that H2A.Z has a conserved function at boundary elements related to nucleosome disassembly both in unicellular and multicellular eukaryotes.

## Materials and methods

### Yeast strains and media

All five null mutants, *hht2∆*, *asp1∆*, *iec1∆∆*, *arp42∆*, *pht1∆*, and *ies2∆* were derived from the version 5 Bioneer library, i.e., a large collection of gene deletion mutants carrying the *kanMX4* cassette marking the gene deletion and *leu1-32 ade6-M216/M210 ura4-D18* auxotrophic markers. The Bioneer strains cannot survive under the absence of nitrogen. Therefore, to produce prototrophic null mutant strains, the Bioneer strains were crossed with the Hu2843 *mat1-M smt0* wild-type strain using standard methods (Ekwall and Thon 2017). The prototrophic mutants produced from each cross were selected using Edinburgh Minimal Medium (EMM) minus leucine, adenine, and uracil and subsequently YES medium containing G418 (150 ug/mL). The resulting mutant strains were named Hu3103 *smt0 hht2∆ kanMX4*, Hu3101 *smt0 asp1∆ kanMX4*, Hu3104 *smt0 iec1∆ kanMX4*, Hu3100 *smt0 arp42∆ kanMX4*, Hu3102 *smt0 pht1∆ kanMX4*, and Hu3113 *smt0 ies2∆ kanMX4*. The epitope tagged H2A.Z (*pht1-myc*) strains were produced from a cross using parental strains from (Buchanan et al. 2009) and Hu2843 resulting in the Hu3110 *smt0 iec1D::ura4 pht1-myc* and the Hu3112 *smt0 pht1-myc* strains.

All strains were grown in semi-solid YES complete media for 2 days 30 ℃ for 48 h and were regrown in liquid Pombe minimal glutamate medium (PMG) + nitrogen in a 200 ml flask to reach 10^6^ cells /ml using a shaking incubator at 200 rpm at 30 °C. Before washing the cells, take 50 ml of culture as time 0 investigation and the rest of culture washed with 200 ml pre-warmed PMG-N and incubated them in 200 ml of PMG-N media then incubated (shaking incubator at 200 rpm at 30 °C).

### Flow cytometry and viability measurements

For flow cytometry analysis (FACS) four time points were considered in this study, T0 (before shift to -nitrogen), T1D (24 h after shift), T1W (1 week after shift) and T2W (2 weeks after shift). For T0 and T1D 50 ml of culture and for T1D and T2W, 100 ml of culture was used. For each time point cells were pelleted and transferred into a 96-round bottom well plate and washed with 200 *∆*l of PBS (centrifuged at 400 g, 5 min, at room temperature) and stained with 150 *∆*l of Live-or-Dye™-Fixable Viability Stain (Biotium, Fremont, CA, USA). This stain was used at 1/1000 dilution in PBS, in the dark, and incubated for 30 min on ice with mild shaking. Then, cells were washed with 200 *∆*l PBS and centrifuged (400 g, 5 min, at room temperature) followed by a fixation step using 200 *∆*l of 70% ethanol and incubated for 30 min on ice in the dark. After washing with 200 *∆*l PBS, cells were incubated for 15 min in 200 *∆*l sodium citrate buffer (50 mM sodium citrate, pH 7.0), washed once with 200 *∆*l sodium citrate buffer, pelleted and resuspended in 200 *∆*l sodium citrate buffer containing 0.2 mg/ml DNAse-free RNase A (Roche diagnostics Scandinavia, Solna, Sweden, 10,109,169,001) and incubated for 3 h at 37 °C. Then, cells were stained with 100 *∆*l of PBS containing 12.5 mg/ml propidium iodide (PI) (Invitrogen AB, Stockholm, Sweden, P4864) by incubating for 30 min at room temperature in the dark. Before FACS analysis, 100 μl of PBS was added into each well and the 96-well plate was immediately analyzed using the multiplex flow-cytometer CytoflexS (Beckman Coulter) and the CytExpert software (www.mybeckman.se). Slow mode running was used to collect and run the samples and the data was recorded based on 20,000 events of live cells in each sample. The total number of cells was selected via forward (FSC) and side (SSC) scattering, and single cells were sorted via FSC vs FSH (height) to exclude doublets. Then, the live cell population was selected via negative signal of Live-or-Dye™ Fixable Viability Staining λEx/λEm 642/662 nm through the FSC-A vs FL3A (R660) channels (FL3A::660A). The DNA content histogram analysis and cell cycle population analysis were performed on live cells population using the signals of PI staining using the gating strategy described in (Zahedi et al. 2020). At the T0 time point, the mononuclear G_2_ cell population was selected through the total area of DNA signal (DNA-A) *vs.* the width of the DNA signal (DNA-W), and in quiescence, G_0_ cells with 1C DNA content were selected via same gating (DNA-A negative, DNA-W negative). A minimal cut-off of 1000 single cells was considered for each sample measurement. The selected data was analyzed via FlowJo software version 9 (https://www.flowjo.com/solutions/flowjo/downloads).

*RNA isolation.*

Wild type and mutant strains were grown in a 200 ml liquid PMG + N medium using a shaking incubator (200 rpm at 30 °C) to reach between 1.0 × 10^6^ and 10 × 10^6^ cells/ml. For each culture, 100 ml was removed for the T0 timepoint, and the rest of the culture was washed with pre-warmed PMG-N and incubated for 24 h in 500 ml of pre-warmed PMG-N using a shaking incubator (200 rpm at 30 °C). For RNA extraction, cells were washed with ice-cold PBS and resuspended in 500 *∆*l of ice-cold RNA extraction buffer (10 mM Tris–HCl pH 8.0, 1 mM EDTA, 2% Triton X-100, 1% SDS, 100 mM NaCl). Then we added 500 µl of Phenol (acidic phenol pH 4.5, Sigma) and 500 µl of glass beads (acid washed, Sigma). The tubes were vortexed vigorously and incubated at 65 °C for 45–60 min. Next, the tube was placed on ice for 5 min and centrifuged (1300 g, 5 min, 4 °C). The upper aqueous part was collected and transferred to a tube with 500 µl of chloroform (Sigma Aldrich), vortexed and centrifuged (1300 g, 5 min, 4 °C). The upper phase was collected and subjected to RNA precipitation at − 20 °C overnight. The precipitated RNA was washed once with 70% ethanol and dissolved in 30 *∆*l H_2_O.

### Chromatin immunoprecipitation sequencing (ChIP-Seq)

Log phase cells grown in PMG or PMG-N media were harvested and cross-linked by 1% formaldehyde for 30 min, and then 125 mM Glycine was added to quench the crosslinking for 5 min. After three time washing with cold PBS, the cells pellet was resuspended in ChIP lysis buffer with 0.5 mm Zirconia/Silica Beads, and then lysed in FastPrep machine for 7 times at max power 6.5. Sonication was done by using Bioruptor® Pico for 10 cycles, and then chromatin concentration was measured with Qubit dsDNA HS assay kit. Immunoprecipitation was performed with 20 µg sheared chromatin, 40 ng spike-in chromatin (activemotif 53,083), 1.6 µl spike-in antibody (activemotif 61,686) and 6 µl anti-c-Myc antibody (Sigma-Aldrich, M4439). After three times washing with low salt wash buffer, high salt wash buffer and LiCl wash buffer successively, ChIP-DNA was extracted by ChIP DNA Clean & Concentrator kit (ZYMO RESEARCH, D5205) and DNA concentration measured by Qubit dsDNA HS assay kit. Sequence library prepared by ThruPLEX DNA-Seq kit (TaKaRa, R400676) with DNA HT Dual Index Kit – 96N Set A (TaKaRa, R400660). Before sequencing, we performed quality control with bioanalyzer high sensitivity DNA analysis, and then the sequencing was performed using the Illumina Nextseq 2000 platform with P3 v3 50 kit (36 + 8 + 8 + 36 cycles, single-end sequencing) at the BEA facility (Huddinge, Sweden).

Raw sequencing data from Nextseq 2000 (Bcl files) were converted and demultiplexed to fastq files using the bcl2fastq v2.20.0.422 program. The STAR 2.7.9a program (Dobin et al. 2013) was used for alignment with *Schizosaccharomyces pombe* reference genome (ASM294v2) and *Drosophila melanogaster* reference genome (dm6). We used *Drosophila* spike in normalization strategy for ChIP-seq data normalization described in (Egan et al. 2016). Samtools was used to count the reads in specific regions, and then we normalized the reads by following spike-in normalization strategy. Data were visualized with the Integrated Genomics Viewer (IGV). For bar diagrams, Microsoft Excel was used to create bar diagrams with unpaired T-test statistics.

### RNA-seq and bioinformatics

To remove rRNA, 3 µg of purified total RNA was treated with Ribominus Eukaryote System v.2 kit (Ambion, Thermo Fisher Scientific). To generate sequencing libraries, a total of 100 ng of rRNA-depleted stocks and Illumina Stranded mRNA Prep Ligation kit (Illumina) were used. To quantify the samples, Qubit (HS dsDNA) was used, and samples were sequenced using an Illumina Nextseq 2000 platform (P3 100 cycle kit, 58 + 58 cycles, paired-end sequencing) at the BEA facility (Huddinge, Sweden) following the manufacturer’s instruction. To normalize samples, ERCC RNA Spike-In Mix 1, dilution 1:100 (Invitrogen, Thermo Fisher Scientific) was added in proportion on the number of vegetative cells in each culture that was used for RNA isolation.

Raw sequencing data from Nextseq 2000 (Bcl files) were converted and demultiplexed to fastq files using the bcl2fastq v2.20.0.422 program. The STAR 2.7.9a program (Dobin et al. 2013) was used to index the Schizosaccharomyces_pombe reference genome (ASM294v2) and the ERCC spike in sequences, and then the resulting fastq files were aligned. The mapped reads were then counted in annotated exons using featureCounts v1.5.1 (Liao et al. 2014). The genome fasta file and annotations (Schizosaccharomyces_pombe.ASM294v2.35.gff3) were obtained from ensembl. The count table from ‘featureCounts’ was imported into the R/Bioconductor program and differential gene expression analysis was performed using the EdgeR package (Robinson et al. 2010). The linear models pipeline of EdgeR was used. For the gene expression analysis, genes that had > 1 count per million in 3 or more samples were used and normalized based only on the ERCC spike in counts using the TMM normalization. To correct for batch effects the second batch with *ies2* samples were normalized at T0 with the average ERCC factor from the first batch.

## Supplementary information

Below is the link to the electronic supplementary material.

Supplementary file1 (XLSX 10922 KB)

## Data Availability

Yeast strains can be requested by writing to KE. The RNA-seq data and the ChIP-seq data have been submitted to the NCBI Gene Expression Omnibus (GEO) under the accession number GSE200378. The processed ERCC normalized RNA-seq data is provided in Supplementary data excel file 1.
